# A Process Evaluation of Intervention Delivery for a Cancer Survivorship Rehabilitation Clinical Trial Conducted during the COVID-19 Pandemic

**DOI:** 10.3390/curroncol30100660

**Published:** 2023-10-16

**Authors:** Courtney J. Stevens, Stephen Wechsler, Deborah B. Ejem, Sarah Khalidi, Jazmine Coffee-Dunning, Jamme L. Morency, Karen E. Thorp, Megan E. Codini, Robin M. Newman, Jennifer Echols, Danielle Z. Cloyd, Sarah dos Anjos, Colleen Muse, Sarah Gallups, Susan C. Goedeken, Kaitlin Flannery, Marie A. Bakitas, Mark T. Hegel, Kathleen Doyle Lyons

**Affiliations:** 1 Department of Psychiatry, Geisel School of Medicine, Dartmouth College, Hanover, NH 03755, USA; 2 Department of Occupational Therapy, School of Rehabilitation Sciences, Massachusetts General Hospital Institute of Health Professions, Boston, MA 02129, USAklyons2@mghihp.edu (K.D.L.); 3 School of Nursing, University of Alabama at Birmingham, Birmingham, AL 35294, USA; 4 Department of Rehabilitation Medicine, Dartmouth-Hitchcock Medical Center, Lebanon, NH 03766, USA; 5 Sargent College of Health and Rehabilitation Sciences, Boston University, Boston, MA 02215, USA; 6 Department of Occupational Therapy, School of Health Professions, University of Alabama at Birmingham, Birmingham, AL 35294, USA; 7 Department of Occupational Therapy, Massachusetts General Hospital, Boston, MA 02114, USA

**Keywords:** neoplasms, rehabilitation, psychosocial oncology, behavioral therapy

## Abstract

The purpose of the present study was to conduct a process evaluation of intervention delivery for a randomized controlled trial (RCT) conducted during the COVID-19 pandemic (NCT 03915548). The RCT tested the effects of a telephone-delivered behavioral intervention on changes in breast cancer survivors’ satisfaction with social roles and activities, as compared to an attention control condition. This process evaluation examined (a) fidelity monitoring scores; (b) participants’ perceived benefit ratings for gaining confidence, reducing distress, adjusting habits and routines, setting goals, and increasing exercise; and (c) field notes, email communications, and transcripts of coach supervision and debriefing sessions. The behavioral and attention control conditions were delivered with a high degree of fidelity (global quality rating score for the BA/PS condition was *M* = 4.6 (*SD* = 0.6) and *M* = 4.9 (*SD* = 0.3) for the attention control condition, where “5” is the highest rating). The behavioral intervention participants perceived greater benefits than the control participants pertaining to goal setting, *t*(248) = 5.73, *p* = <0.0001, adjusting habits and routines, *t*(248) = 2.94, *p* = 0.0036, and increasing exercise, *t*(248) = 4.66, *p* = <0.0001. Moreover, coaches’ perceptions regarding the behavioral intervention’s therapeutic aspects aligned with the study’s a priori conceptual model including the use of a structured process to set small, observable goals and facilitate the independent use of problem-solving skills. However, coaches also noted that aspects of the attention control condition, including the perceived relevance of the educational content and opportunities for social support, may have made it more therapeutically potent than intended. The pandemic may have affected the activity goals behavioral intervention participants could set as well as augmented the relevancy of social support provided in both conditions.

## 1. Introduction

While most cancer survivors recover steadily after primary treatment, approximately one-third report participation restrictions within the five years post treatment [1]. The term participation restriction refers to the inability to fully engage in valued activities and fulfill roles to one’s satisfaction [2]. A recent meta-analysis found that challenges with daily activities were one of the top unmet supportive care needs of breast cancer survivors (reported by 54% of breast cancer survivors), along with the need for social support (reported by 74% of breast cancer survivors) and information support (reported by 45% of breast cancer survivors) [3].

While decreasing participation restrictions is an explicit goal of rehabilitation, the evidence base for how to best facilitate this in the context of cancer survivorship is sparse [4,5]. Recent pilot studies have used self-management and metacognitive strategy training approaches to help women perform daily activities to their satisfaction [6,7]. Our team developed an intervention that uses the structure and philosophy of Behavioral Activation (BA) and Problem-solving (PS) Treatment [8,9] to help female breast cancer survivors set and achieve goals related to activity engagement [10,11], thereby reducing participation restrictions. We referred to this intervention as BA/PS. We chose to deliver the intervention by telephone because barriers related to time and geography limit the uptake of rehabilitative care services in oncology [12,13]. In late 2019, we began a randomized controlled trial (RCT) comparing the BA/PS intervention to an attention control condition [14]. The primary outcomes of the trial were the Patient-Reported Outcomes Measurement Information System (PROMIS) social participation scales [15,16].

When the COVID-19 pandemic disrupted research across the United States [17], our team discussed the implications for our study. We recognized that pandemic-related impacts on daily life, such as social distancing mandates, might exacerbate participation restrictions and affect how each condition might be experienced. However, we felt the intent of the BA/PS intervention was still important because breast cancer survivors still needed to find ways to accelerate recovery and maximize well-being through meaningful activity engagement despite constraints created by the pandemic. Furthermore, during the pandemic, rates of loneliness, anxiety, depression, and stress were on the rise among breast cancer survivors [18] while, at the same time, participation in key health behaviors such as physical activity was declining [19,20]. Thus, the pandemic set the stage for an even greater need to address participation restrictions among breast cancer survivors. As such, we proceeded with the trial.

Before the primary data collection and analysis of the RCT were completed, we conducted a process evaluation of intervention delivery. Process evaluations provide an opportunity to explore the context, theoretical basis, and stakeholder perceptions of complex interventions to identify key uncertainties to be addressed by future research [21]. In the context of the COVID-19 pandemic, conducting a process evaluation seemed even more important. Before we could interpret the main outcomes of the trial, we needed to explore the fidelity of intervention delivery and the degree to which participants perceived benefits. Given that the BA/PS intervention aimed to maximize social participation at a time when society was encouraged to adhere to “social distancing,” the process evaluation was also expected to help us more keenly understand how it was experienced by coaches and participants, relative to the attention control condition. Our process evaluation answers the following questions: (1) To what degree were the BA/PS and attention control conditions delivered with fidelity to the protocol? (2) To what degree did participants perceive benefit from the BA/PS and attention control conditions? (3) What were the impressions of the coaches who delivered the interventions regarding the therapeutic potential of both conditions, particularly in the context of the COVID-19 pandemic?

## 2. Materials and Methods

### 2.1. Overview

**Figure 1:**
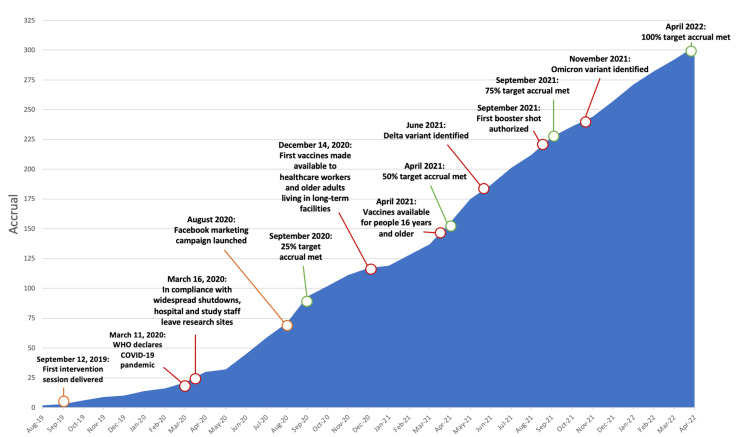
Accrual timeline.

This paper describes a process evaluation of intervention delivery within an RCT (NCT 03915548). Details regarding the protocol [14] and the recruitment strategy utilized during the COVID-19 pandemic [22] are published elsewhere. Figure 1 depicts the timeline of trial activities and pandemic milestones. We collected data for this analysis prior to finishing primary data collection in March of 2023.

### 2.2. Description of Intervention

Both conditions involved nine telephone sessions with a coach who was either an occupational therapist (*n* = 9) or a nurse (*n* = 1). The first six sessions occurred weekly, while the last three sessions occurred monthly. Each participant worked with the same coach for all nine sessions, with rare exceptions (i.e., coach began medical leave or parental leave).

#### 2.2.1. Behavioral Activation/Problem-Solving (BA/PS)

The intervention is informed by the structure and principles used in the therapies of Behavioral Activation [8] and Problem-solving Treatment [9]. The BA/PS intervention was designed to help people increase activity engagement in self-identified, role-relevant activities that promote well-being and recovery. Participants were mailed a workbook that contained educational handouts and goal-setting worksheets. Session 1 included education regarding the purpose of the intervention, the ways in which activities contribute to health and well-being, and the importance of a positive problem orientation when tackling cancer-related challenges. Session 1 concluded with elicitation of the participants’ history and priorities, utilizing the Canadian Occupational Performance Measure [23].

**Figure 2:**
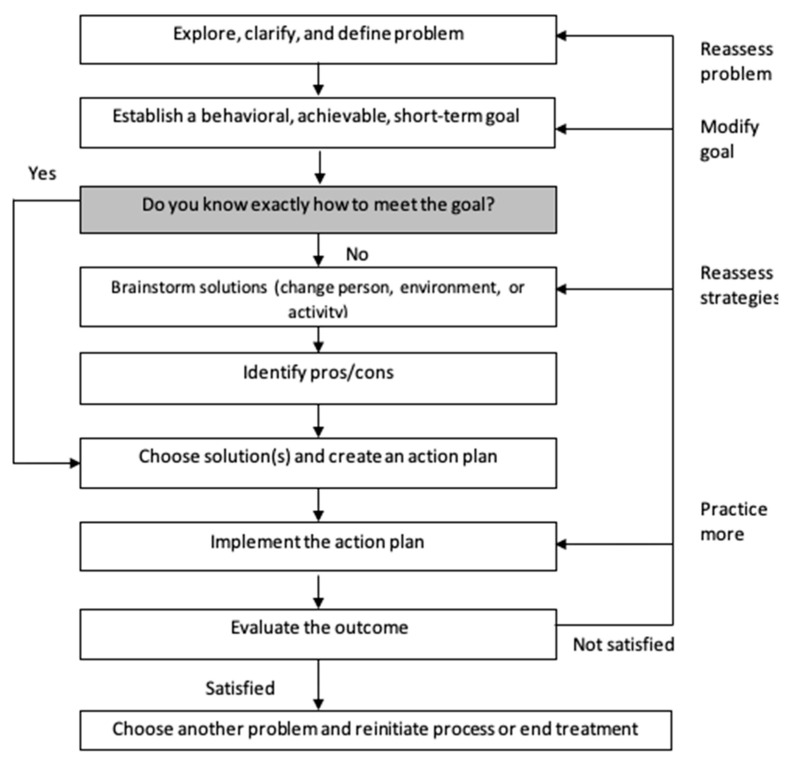
Behavioral Activation/Problem-Solving session structure. Note. Figure 2 was previously included in a protocol paper describing the BA/PS intervention (see [14]).

Session 2 included information regarding energy management and introduced a method to consider the “who, when, where and how” of activity engagement to maximize enjoyment and ease of performance. During sessions 2–9, coaches guided participants through a goal-setting framework to address at least one activity that they would like to engage in during the coming week. Coaches guided participants through the framework each week, asking clarifying questions, as necessary, to maximize the safety and feasibility of participants’ action plans. The steps of the process are depicted in Figure 2.

#### 2.2.2. Attention Control

The comparison condition was designed to control for the non-specific therapeutic effects of warm, supportive attention from the coach. Because we wanted a similar “dose” of contact between conditions, we wanted to offer a credible attention control condition that had the potential to meaningfully engage participants. As such, we used education about cancer survivorship topics to structure the attention control sessions. To mirror the BA/PS condition, in which participants were given the autonomy to choose activities to discuss and target with the weekly goal-setting exercises, participants in the attention control condition were given a menu of educational topics to choose from for review and discussion at each session (e.g., exercise, healthy nutrition, fatigue management, cognitive side effects).

**Figure 3:**
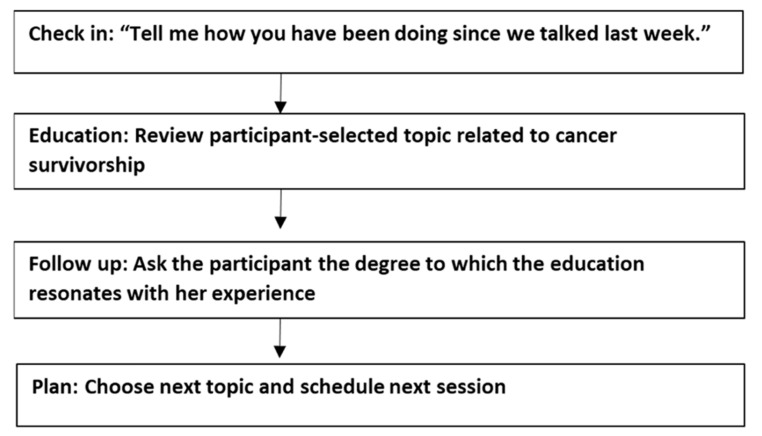
Attention control session structure.

The steps of each weekly session are depicted in Figure 3. The bulk of the session focused on reviewing educational content found on the Springboard Beyond Cancer website (https://web.archive.org/web/20201016160255/, https://survivorship.cancer.gov/springboard, accessed on 8 October 2023). Participants were not provided with a workbook, but they were given the web address if they wanted to review the information during or after the session. Coaches were instructed to avoid directly asking participants how they would apply the information, as goal setting and action planning are behavior-change techniques hypothesized to drive effects of the BA/PS intervention [24]. It should be noted, however, that the educational content was designed to foster disease self-management and offers direct suggestions of activities that may help to address concerns experienced by many survivors of cancer.

### 2.3. Participants

Individuals were eligible for the study if they (a) were over the age of 18; (b) completed treatment for Stage I, II, or III breast cancer within the past year; and (c) reported difficulty performing daily activities as defined as a score of 10 or greater on the Work and Social Adjustment Scale [25]. Those interested in participating were excluded if they did not speak English, had non-correctable hearing loss, had moderate-severe cognitive impairment as indicated by a score of less than three on a six-item cognitive screener [26], or had a history of severe mental illness (i.e., schizophrenia, bipolar disorder), current major depressive disorder, active suicidal ideation, or active substance misuse self-reported or documented within the medical record.

### 2.4. Coaches

A total of 10 coaches delivered the intervention and control conditions. For the first two years, seven occupational therapists (J.L.M., M.E.C., R.M.N., K.E.T., S.d.A., J.E., D.Z.C) delivered the intervention and control conditions. In the final nine months of the study, two additional occupational therapists (C.M. and S.C.G) were trained to deliver the intervention and control conditions, and one nurse (S.G) was trained to deliver the control condition. The coaches were all female and had a mean of 9.9 years (*SD* = 5.7; range 5–19) of clinical practice prior to starting the study. Only two had clinical experience working in oncology rehabilitation prior to the study. Consistent with the Occupational Therapy Practice Framework of the American Occupational Therapy Association, all of the occupational therapists had extensive experience teaching people how to maximize their personal capacity, adapt activities, and modify environments in order to improve a person’s ability to do their valued activities [27].

### 2.5. Sources of Data

#### 2.5.1. Fidelity Ratings

Telephone sessions were audio-recorded with the permission of participants and uploaded to a secure site. Fidelity monitoring occurred throughout the course of the study using rating sheets developed specifically for each condition. The ratings evaluated either (a) the presence or absence of the prescribed and proscribed elements of each condition or (b) the quality with which the element was delivered. The BA/PS rating sheet had 19 items, and the attention control condition had 13 items.

Fidelity monitoring was conducted by a licensed psychologist (M.T.H.) who co-developed the intervention and control conditions. The plan was to review two sessions from 15% of the sample of each condition (2 sessions from 23 women in each condition, for a total of 92 sessions). For the first three months of the study, the coaches could also request monitoring for sessions where they wanted feedback on their delivery. The final number of sessions rated included 58 BA/PS sessions and 55 control condition sessions. Sixty-two participants and all 10 coaches were represented in this data set.

#### 2.5.2. Perceived Benefits

As part of the outcome assessment battery, coordinators blinded to treatment conditions administered five questions regarding the perceived benefit of the telephone sessions. The timing of administration was selected to coincide with the transition from the more time-intensive 6 weekly sessions to the monthly follow-up sessions. Using a three-point scale scored “not at all” = 0, “some” = 1, or “a great deal” = 2, participants were asked to rate the degree to which the phone sessions helped them to (a) gain confidence, (b) reduce distress, (c) adjust habits and routines, (d) set goals, and (e) increase exercise. The selection of these perceived benefits was based on our team’s conceptual model of outcomes [14] and prior pilot data [11,28]. The questions were adapted from a clinical trial of a comparable biobehavioral intervention for older adults [29,30].

#### 2.5.3. Feedback from Coaches and Participants

The principal investigator (PI; K.D.L.) kept field notes documenting feedback to and from coaches during the initial training to deliver each condition and weekly 1 h group supervision sessions. Email communications about intervention delivery were also pasted into the field notes.

Two audio-recorded debriefing sessions (facilitated by K.D.L. and M.T.H.) were held over Zoom one month after intervention delivery ended for all participants. This analysis focused on the primary goal of the debriefing sessions, which was to explore coaches’ perceptions of the aspects of each condition that may have been therapeutic for participants as well as how the context of the pandemic was featured in conversations with participants. Eight of ten coaches participated in the debriefing sessions; the remaining coaches provided feedback via email. The recordings were transcribed and proofread by graduate students.

### 2.6. Analysis

Descriptive statistics were used to summarize the fidelity ratings and perceived benefit scores. We used chi square tests for categorical data and independent samples *t* tests for continuous data to determine if there were differences in the fidelity ratings and perceived benefit scores between groups. We considered *p* values of <0.05 to indicate a statistically significant difference between conditions.

The PI performed a content analysis of the field notes and debriefing transcripts using the following process as described by Bachiochi and Weiner [31]. After repeatedly reading the transcripts and field notes, a coding scheme was iteratively developed and applied to categorize feedback from the coaches. For example, codes like “people enjoyed the attention”, and “helped people to see small progress” were used to categorize examples in the transcripts. To maximize the credibility of our interpretations [32], coaches reviewed the results and were asked to (a) verify that the code reflected the quotations listed for each code and (b) indicate whether they agreed, disagreed, or neither agreed nor disagreed with the sentiment expressed in the code. This feedback was often supplemented by additional examples of the code or comments that provided nuance (e.g., “I only heard that from one or two of my participants”). The feedback was used to refine the findings and supporting quotations, and all coaches read and approved the final version of the results.

## 3. Results

### 3.1. Participants

The characteristics of our sample are reported in detail elsewhere [22]. In brief, 303 women enrolled, and 284 completed baseline procedures and were subsequently randomized to condition (*n* = 144 BA/PS and *n* = 140 attention control). The mean age for the sample was 56.1 (*SD =* 10.2) years. Most participants were White (82%), employed full-time (54%), married (66%), and had a college education (68%). Most participants had either stage 1 or 2 disease (82%). At the conclusion of the study, 83% of participants randomized to BA/PS (120/144), and 85% of participants randomized to attention control (119/140) completed all 9 study intervention sessions.

### 3.2. Fidelity Ratings

The fidelity rating scores are presented in Table 1. Overall, the ratings for both conditions were high, with the majority of items having a mean score >4 on a rating scale where the highest score was 5. The global quality rating score for the BA/PS condition was *M* = 4.6 (*SD* = 0.6) and *M* = 4.9 (*SD* = 0.3) for the attention control condition. This difference was statistically significant (*t* = 2.97, *p* = 0.004) but not clinically meaningful.

The lowest scores were seen for the item that rated whether the coaches set an agenda for the session, occurring 28% of the time during the BA/PS sessions and 51% of the attention control sessions. We examined the data to assess whether the lower scores appeared to be driven by a certain set of coaches, but there did not appear to be a pattern.

The fidelity scores were slightly higher for the attention control sessions compared to the BA/PS sessions. The BA/PS items with mean scores less than 4 were those that evaluated more complicated, process-oriented steps such as evaluating and processing satisfaction with goal attainment and communicating the intervention rationale in response to goal attainment or non-attainment. While the coaches adhered well to the attention control protocol to avoid setting goals, brainstorming, or action planning, one comment from the fidelity monitor is notable: “*The coach provided such an enthusiastic and supportive review of last week’s activities that it almost bordered on the debriefing step of BA/PS*”.

### 3.3. Perceived Benefit

Table 2 displays the number of participants in each condition who reported that the intervention they received helped them to gain confidence, reduce distress, adjust habits and routines, set goals, and increase exercise either “not at all,” “some,” or “a great deal,” respectively. Between-group differences were observed such that BA/PS participants more strongly endorsed intervention benefits for goal setting (*M* = 1.7, *SD* = 0.5 vs. *M* = 1.3, *SD* = 0.7), *t*(248) = 5.73, *p* = <0.0001, adjusting habits and routines (*M* = 1.5, *SD* = 0.6 vs. *M* = 1.3, *SD* = 0.7), *t*(248) = 2.94, *p* = 0.0036, and increasing exercise (*M* = 1.5, *SD* = 0.6 vs. *M* = 1.1, *SD* = 0.7), *t*(248) = 4.66, *p* = <0.0001. Effect sizes for these statistically significant tests ranged from small, Cohen’s *d* = 0.37, for adjusting habits and routines, to medium, Cohen’s *d* = 0.73, for goal setting, and Cohen’s *d* = 0.59 for increasing exercise. Between-group differences were null for gaining confidence (*M* = 1.6, *SD* = 0.5 vs. *M* = 1.5, *SD* = 0.6), *t*(248) = 1.41, *p* = 0.16, and reducing distress (*M* = 1.4, *SD* = 0.6 vs. *M* = 1.3, *SD* = 0.7), *t*(248) = 0.92, *p* = 0.36.

### 3.4. Feedback from Coaches

Content analysis of field notes and debriefing transcripts revealed several aspects of the BA/PS intervention and attention control conditions that coaches perceived as potentially therapeutic. In both study conditions, coaches described developing a strong rapport with participants and feeling a sense of privilege witnessing participants’ struggles and supporting their successes: “*…we [coaches] all…seemed to have very similar experiences of connectedness with the participants…we had the time to listen to these women, to hear about every aspect of their life… (Coach 06)*”.

#### 3.4.1. Behavioral Activation/Problem-Solving (BA/PS)

Coaches identified the following BA/PS features as particularly potent: (a) a structured process to approach challenges with creativity; (b) the importance placed on making small and observable progress toward goals; and (c) the translation of skills through participant-directed problem-solving and reflection.

##### Structured Process to Approach Challenges with Creativity

Coaches perceived introducing participants to a positive problem orientation to be a critical component of the BA/PS intervention. “*I realized over time the importance of framing the intervention and introducing them to the optimistic kind of orientation to solving problems… that mindset is an integral part of the intervention. (Coach 01)*”. This positive problem orientation paired with goal setting was a powerful experience for many participants as Coach 06 described: “*It’s a very simple process, but I think it’s very effective when they’re [the participant is] having… a hard time thinking through things, when they have brain fog, when they just feel stuck and like they don’t know how to get through something that typically has been simple for them [in the past], so giving them that tool…it was very helpful*”.

##### Making Small and Observable Progress toward Goals

The BA/PS intervention structure and worksheets provided concrete tools that could show progress. Coaches frequently described “helping people see and acknowledge progress, get away from black and white thinking of they either ‘fail or succeed’, ‘do everything or do nothing’, and they [the coaches] talk a lot about the value of partial solutions… (PI notes from supervision)”. Coaches perceived this reframing and the ability to support participants to observe progress toward goals as potentially therapeutic: “it was very helpful, going through that with the women…even if they didn’t meet their goal, just seeing them make progress and being able to encourage them through that in such a visual way that they can look at [on their worksheets]. (Coach 06)”.

##### Translation of Skills through Participant-Directed Problem-Solving and Reflection

A key feature of the BA/PS intervention is participant-directed problem-solving, i.e., coaches provide a structure for brainstorming, but solutions are generated by participants. Coaches felt that this aspect of the intervention helped participants gain confidence, a sense of control, and the ability to generalize problem-solving skills to other aspects of their lives: “*It is quite beneficial and all the more rewarding for the participant when they feel they have come to a conclusion/solution on their own. (Coach 03)*”. Coach 07 echoed this benefit as she observed participants generalize BA/PS skills: “*…the goal setting approach became a skill instead of a strategy, I could see that being transferred in so many other situations and other contexts from these women’s lives*”. This led to feelings of empowerment: “*…they have control of the situation, way more than they believed they had… when they are empowered and use that in different contexts…They are not depending on other people to guide them…they have control of it (Coach 07)*”. Feelings of empowerment and translation of skills were further fostered by providing participants with time to reflect. “*I found [step 1] to be really important, asking them the questions, ‘What’s worked well in the past and what didn’t work well?’ I think it helped set up the future problem solving…that helped a lot. (Coach 02)*”.

While the coaches generally felt that the BA/PS intervention was relevant and helpful, they also identified some barriers to its implementation. First, there were participants who were unfamiliar with or uninterested in goal setting. The supervision field notes detailed one week where the discussion focused on Coach 04 having a “string of participants” who were resistant to the utility of identifying a weekly activity goal and action planning to maximize chances of meeting the goal. Another participant told the coordinator collecting outcome data that she expected the intervention would be more directive and tell her what to do to foster recovery, instead of asking her to set her own goals. Another challenge was that sometimes the BA/PS structure could elicit details about depressive symptoms or other intractable symptoms experienced by participants. Coach 05 felt that she “*wasn’t prepared…for sometimes how [the BA/PS intervention] would flesh out more things that I didn’t feel equipped to handle…a lot of people had issues with depression…I really didn’t know how to get them back to what we were working on*”.

#### 3.4.2. Attention Control

Coaches identified the following features of the attention control condition as particularly potent or therapeutic: (a) the perceived relevance and utility of the educational content, and (b) the opportunity for social contact and support.

##### Perceived Relevance, Utility, and Timeliness of Educational Content

Many attention control participants appeared to find the educational content useful and relevant, as Coach 01 said some participants would say things like, “*Wow, this is really helpful, thank you so much, I’m learning so much*”. For some participants, “…*this was all new information and they found it really, really helpful… (Coach 02)*”. Participants seemed to appreciate being able to decide upon the topic of educational content for each session: “*People, overwhelmingly, it sounded like, liked that they could choose the topics. (Coach 01)*”. Conversely, Coach 01 also described some participants already seemed familiar with the educational topics: “*…there [were] some outliers that were highly intelligent, highly studied, and had received this kind of information already and were just going along with you*”. In these instances, participants appeared to view the attention control sessions primarily as an opportunity to provide feedback regarding the information. We did not discourage this as the final step in the attention control session protocol was to ask participants to reflect on the degree to which the information mirrored their experience.

##### Opportunity for Social Support and Contact

Coaches perceived participants as generally appreciating the opportunity for social support and contact provided by the attention control sessions. “*I was concerned that, you know, my personality could be affecting their reaction…a lot of the [Attention Control] participants that I had really enjoyed the education. And I didn’t know if it was because they were using it as a pseudo-therapy session… [maybe] they really enjoyed it just because they liked talking to their coach. (Coach 05)*”. The attention may have been particularly potent during the pandemic when the coaches reported that many of their participants were feeling isolated: “*…a lot of them were isolated… Rural isolation and also during the pandemic…a lot of people enjoyed this [telephone sessions] for the support… (Coach 05)*”.

#### 3.4.3. Context of a Clinical Trial during a Pandemic

Throughout the study, coordinators collecting the outcome measures reported that participants in both groups endorsed challenges with answering PROMIS social participation questions due to restrictions during the pandemic on social gathering and other regularly scheduled events providing socialization. Participants noted external limitations on what they could do, and this was echoed by the coaches: “*You know, for the pandemic, say their goal was exercise; there were so many barriers. Like, ‘oh, well, I used to go to the gym and swim in the pool, but now I’m not comfortable going to the gym.’ (Coach 02)*”. Conversely, other participants reported that they had more time to work on self-care goals during the pandemic: “…*some people were able to designate the time to…setting goals and things because they were maybe not working, or working from home or remotely…When things started opening up again, it felt like people were busier and had a harder time staying as consistent with the goal-setting… (Coach 01)*”.

Unrelated to the pandemic, coaches noted study attrition may have been mitigated by a potential research procedural artifact. Specifically, our team continued to contact participants unless and until they told us they wanted to withdraw from the study. “*I think there is a difference [between research and clinical practice]…If you go to therapy, and you miss therapy twice, you’re done, right? You lose your spot… But for us, we kept trying to contact them in different ways: texting, calling, emailing…I think this also reinforced their impression of, ‘I’m important, they really care about me.’… many [participants] told me, ‘Thank you so much for persisting to contact me, don’t give up on me.’ Coach 07*”.

## 4. Discussion

The purpose of this process evaluation was to explore data allowing us to determine the extent to which the BA/PS and attention control conditions were delivered with fidelity, to explore stakeholder perceptions of intervention delivery to better understand the results of the RCT (under analysis), and to identify considerations for future research. The context of our RCT occurring during the COVID-19 pandemic made conducting this process evaluation even more necessary. A recent survey study of investigators conducting clinical trials during the pandemic found that 69% experienced challenges with conducting their ongoing trials, 78% believed COVID-19 had an impact when initiating new trials, and 63% were forced to halt recruitment on open trials [17]. We kept our trial open by modifying our recruitment strategy [22], but the environmental and social context during the bulk of our recruitment period was entirely different from when the research was funded [33,34,35]. The results of this process evaluation suggest the pandemic may have influenced the potential therapeutic effects of both conditions.

### 4.1. Fidelity Ratings

The high global scores for both the BA/PS and attention control conditions indicate that the interventions were delivered with high fidelity to protocol. Despite our concern that we might need to make modifications to account for the pandemic, we were able to deliver the conditions as designed. While other intervention studies had to modify their protocols for telehealth delivery [36,37] or omit certain features [38], our team had already determined pre-pandemic that phone-delivery would allow us to reduce access barriers for rural participants. This turned out to be a prophetic choice. Further, the structure of both conditions was such that content was tailored to participants’ stated interests and challenges session to session. This flexibility also likely contributed to our ability to deliver the interventions as planned.

Fidelity items being generally over a mean of 4 indicates an overall strong quality of delivery by the coaches. It is not surprising that the fidelity scores are slightly lower for BA/PS as coaches had the option to request monitoring of challenging BA/PS sessions (to receive feedback). Additionally, BA/PS had more treatment components that were scored than the attention control condition. Lower scores for agenda setting in the BA/PS condition is harder to explain; it is possible that agenda setting may have sometimes occurred prior to coaches starting the recorder and, therefore, was not captured. Regardless, the mean scores for the BA/PS condition were generally above 4.5. Process items had lower scores, but they required a higher level of skill and clinical reasoning, making them more challenging than other items to deliver. Indeed, the field notes from weekly supervision sessions illustrate these challenges, as the coaches found that the BA/PS structure revealed intractable problems and sometimes necessitated more education about the intervention rationale to help participants see the value in weekly goal setting. For this reason, it may be most useful to implement this type of intervention within the context of a multidisciplinary clinical team. Overall, the study was able to be conducted with high treatment fidelity, comparable to fidelity scores obtained for the delivery of other complex interventions in oncology [39,40], despite being conducted in the context of the pandemic.

### 4.2. Perceived Benefit

On average, participants in both conditions endorsed “some” to “a great deal” of benefit for all benefits assessed. Statistically significant between-group differences, small to medium in magnitude, were observed for adjusting habits and routines, increasing exercise, and goal setting, with BA/PS participants perceiving the greatest benefits. These findings are encouraging as adjusting habits and routines and goal setting are the hypothesized primary mechanisms of action through which BA/PS impacts participation, per our conceptual model [14].

Furthermore, our past research [28,41,42] has consistently demonstrated an interest from participants in using the BA/PS intervention to help them increase time spent exercising during cancer treatment recovery, and the strong scores for the perceived benefit of BA/PS on increasing exercise are consistent with these past findings. This finding may inform future research as it suggests that a BA/PS intervention for reducing participation restrictions could be used to help survivors of breast cancer increase exercise, even if increasing exercise is not an explicit focus of the study framing or intervention content. Physical inactivity is a modifiable risk factor for breast cancer recurrence [43], but exercise tends to decline post-treatment [44,45]. Thus, future exercise trials targeting survivors of breast cancer might consider adopting the BA/PS intervention framework.

### 4.3. Coach Feedback

With respect to the BA/PS condition, the coaches’ perceptions regarding the therapeutic aspects of the intervention generally mapped onto our team’s conceptual model (see [14]). Specifically, elements of adaptive coping (“approaching challenges with creativity and an open mind”) and goal adjustment (“making small and observable progress toward goals”) helped to foster greater participation via skill application (“participant-directed problem-solving and reflection”). This supports confidence that the BA/PS participants received an intervention that achieved its intended functions. Conversely, it is notable that the education content provided to the attention control participants contained suggested actions participants could take with respect to the educational topic they were learning about. This content may have overlapped more with the goal setting and action planning content of the BA/PS intervention than intended, perhaps contributing to the high perceived relevance and utility of educational content expressed by the attention control participants.

In one study examining behavior change during the pandemic with adults in the United States, increased available time was the most commonly cited reason for both positive and negative behavior changes [46]. This is consistent with observations from coaches in the current trial noting the pandemic’s impact on both opportunities and limitations for participants. For example, increased social restrictions afforded an increase in daily leisure time that expanded possibilities for how some participants could structure their days—perhaps yielding more time to absorb and reflect on the educational materials for the attention control participants and more time to engage in planned goal pursuits for the BA/PS participants. At the same time, social restrictions clearly constrained the range of goal targets possible for BA/PS participants, as noted by both participants and coaches. Another study indicated that step counts and moderate-to-vigorous physical activity decreased among breast cancer survivors when mandates enforcing confinement were instituted during the pandemic [47]. As such, it appears that the pandemic had idiosyncratic effects on participation restrictions related to participants’ social and environmental contexts during this time.

Efforts by the study team to manage attrition through persistent outreach likely contributed to the high rates observed for study session completion across both conditions (87% of those who started the intervention, i.e., attended session 1, subsequently went on to complete all 9 sessions). Beyond keeping enrollment numbers high, outreach efforts may have provided an important source of social support and contact with the outside world during a time when loneliness and social isolation were on the rise among cancer survivors [18] and in the broader population writ large [48].

We can only speculate as to how outreach efforts may have contributed to participants’ sense of alliance with coaches, whether intentionally (for BA/PS participants) or unintentionally (for attention control participants). It appears possible that the pandemic fostered greater receptivity to the attention control condition than would have occurred when access to resources and social connection opportunities were less scarce. While BA/PS participants also expressed sentiments of social support, the difference is that “acting like a coach” and conveying social support are intended qualities of the intervention delivery style [49]; thus, if participants in the attention control condition were also perceiving heightened degrees of social commitment and encouragement from the coaches, that may have fostered a sense of alliance that extended beyond the intended effect of time-matched contact, thereby increasing the potency of the control condition.

### 4.4. Limitations

Analyses for this investigation were conducted by members of the study team and PI who have a vested interest in ensuring success from running the intervention and collecting outcome data. To maximize credibility [32] and minimize potential bias, content analysis data were collected prior to the analysis of the trial’s primary outcomes. Member checking was used to show codes and quotations and allow coaches to provide clarification, as needed. Further, we specifically searched for “negative cases” or things that were voiced by fewer participants to help us consider multiple viewpoints and triangulate perspectives from participants and coaches. Herein, we have attempted to present examples of less favorable sentiments as examples of various unintended effects arising from both conditions and the historical timeframe during which the data were collected.

## 5. Conclusions

This process evaluation determined both the BA/PS and attention control conditions were delivered with a high degree of fidelity to the protocol. Furthermore, participants in both conditions endorsed intervention benefits for all areas assessed, but BA/PS participants more strongly endorsed benefits pertaining to goal setting, adjusting habits and routines, and exercising, with the largest between-group effect observed for goal setting. Finally, coaches perceived therapeutic aspects of intervention components for both conditions. Intervention aspects perceived as most therapeutic for BA/PS mapped on well to our a priori conceptual model; however, there may have been aspects of the attention control condition that made it more potent than intended, particularly as delivered during the COVID-19 pandemic.

## Figures and Tables

**Table 1:** Fidelity scores.

All Items	BA/PS (*n* = 58)		Attention Control (*n* = 55)		
	*n* (%)	Mean (*SD*; Range)	*n* (%)	Mean (*SD*; Range)	*p*
***Items pertaining only to session 1***	***n* = 5**		***n* = 2**		
Review study purpose	5 (100)		2 (100)		--
Review session structure	5 (100)		2 (100)		--
Problem-solving attitude	5 (100)		N/A		--
Activity review/COPM	5 (100)		N/A		--
Review structure and rationale for BA/PS	5 (100)		N/A		--
***Items for all sessions***	***n* = 58**		***n* = 55**		
Global rating		4.6 (0.6; 2–5)		4.9 (0.3; 4–5)	0.004
Set agenda	19 (28)		27 (51)		0.02
Review results of action plan from previous week		4.7 (0.6; 3–5)		N/A	--
Rated satisfaction with effort and outcome and reviewed BA/PS rationale		3.8 (1.1; 0–5)		N/A	--
Defined new activity challenge		4.9 (0.5; 2–5)		N/A	--
Established realistic goal		4.7 (0.8; 0–5)		N/A	--
Generated alternative solutions		4.6 (0.9; 3–5)		N/A	--
Implemented decision-making guidelines and choosing the solutions (pros and cons)		3.2 (1.1; 2–5)		N/A	--
Created action plan		4.9 (0.8; 0–5)		N/A	--
Process (teaching tasks)		3.5 (1.0; 2.5)		N/A	--
Communication and interpersonal effectiveness		5.0 (0.3; 3–5)		N/A	--
Reviews education and recommendations		5.0 (0; 0) ^a^		4.9 (0.7; 0–5)	0.45
Asks how education is relevant to participant’s life		4.6 (1.3; 1–5) ^a^		4.8 (1.0; 0–5)	0.58
Refer to MD or specialist if wanted		5.0 (0; 0) ^b^		5.0 (0; 0) ^c^	--
Reviewed previous week activities	N/A		45 (90)		--
Avoids components of BA/PS: - Avoids goal setting		N/A		5.0 (0; 0)	--
- Avoids brainstorming		N/A		5.0 (0; 0)	--
- Avoids action planning		N/A		5.0 (0.2; 4–5)	--

Note. N/A = not applicable; COPM = Canadian Occupational Performance Measure [23] ^a^ Only occurred in 10 of the sessions ^b^ Only occurred in 1 session ^c^ Only occurred in 3 sessions.

**Table 2:** Perceived benefits of the BA/PS and control conditions.

	Total (*n* = 250)		BA/PS intervention (*n* = 123)		Attention control (*n* = 127)	
	*n*	(%)	*n*	(%)	*n*	(%)
**Perceived Benefit Questionnaire (PBQ)**						
Gain Confidence						
0—Not at all	12	4.8	3	2.4	9	7.1
1—Some	97	38.8	47	38.2	50	39.4
2—A great Deal	141	56.4	73	59.3	68	53.4
Reduce Distress						
0—Not at all	24	9.6	10	8.1	14	11
1—Some	110	44	53	43.1	57	44.9
2—A great Deal	116	46.4	60	48.8	56	44.1
Set Goals						
0—Not at all	17	6.8	3	2.4	14	11
1—Some	85	34	26	21.1	59	46.5
2—A great Deal	148	59.2	94	76.4	54	42.5
Adjust Habits and Routines						
0—Not at all	22	8.8	8	6.5	14	11
1—Some	106	42.4	43	35	63	49.6
2—A great Deal	122	48.8	72	58.5	50	39.4
Increase Exercise						
0—Not at all	33	13.2	9	7.3	24	18.9
1—Some	117	46.8	48	39	69	54.3
2—A great Deal	100	40	66	53.7	34	26.8

## Data Availability

The qualitative and quantitative datasets used in the analyses generated for the current study are available from the last author (KDL) on reasonable request.

## References

[1] Ness K.K., Wall M.M., Oakes J.M., Robison L.L., Gurney J.G. (2006). Physical performance limitations and participation restrictions among cancer survivors: A population-based study. Ann. Epidemiol..

[2] World Health Organization (2002). International Classification of Functioning, Disability, and Health (ICF).

[3] Fan R., Wang L., Bu X., Wang W., Zhu J. (2023). Unmet supportive care needs of breast cancer survivors: A systematic scoping review. BMC Cancer.

[4] Egan M.Y., McEwen S., Sikora L., Chasen M., Fitch M., Eldred S. (2013). Rehabilitation following cancer treatment. Disabil. Rehabil..

[5] Loh S.Y., Musa A.N. (2015). Methods to improve rehabilitation of patients following breast cancer surgery: A review of systematic reviews. Breast Cancer (Dove Med. Press).

[6] Loubani K., Schreuer N., Kizony R. (2022). Telerehabilitation for Managing Daily Participation among Breast Cancer Survivors during COVID-19: A Feasibility Study. J. Clin. Med..

[7] Boland L., Bennett K., Cuffe S., Gleeson N., Grant C., Kennedy J., Connolly D. (2019). Cancer survivors’ experience of OptiMal, a 6-week, occupation-based, self-management intervention. Br. J. Occup. Ther..

[8] Hopko D.R., Lejuez C.W., Ruggiero K.J., Eifert G.H. (2003). Contemporary behavioral activation treatments for depression: Procedures, principles, and progress. Clin. Psychol. Rev..

[9] Hegel M.T., Arean P.A. (2003). Problem-Solving Treatment for Primary Care: A Treatment Manual for Depression, Project IMPACT.

[10] Hegel M.T., Lyons K.D., Hull J.G., Kaufman P., Urquhart L., Li Z., Ahles T.A. (2011). Feasibility study of a randomized controlled trial of a telephone-delivered problem-solving-occupational therapy intervention to reduce participation restrictions in rural breast cancer survivors undergoing chemotherapy. Psychooncology.

[11] Lyons K.D., Hull J.G., Kaufman P.A., Li Z., Seville J., Ahles T.A., Kornblith A.B., Hegel M.T. (2015). Development and initial evaluation of a telephone-delivered, Behavioral Activation and Problem-solving Treatment Program to address functional goals of breast cancer survivors. J. Psychosoc. Oncol..

[12] Cheville A.L., Kornblith A.B., Basford J.R. (2011). An examination of the causes for the underutilization of rehabilitation services among people with advanced cancer. Am. J. Phys. Med. Rehabil..

[13] Stubblefield M.D. (2017). The Underutilization of Rehabilitation to Treat Physical Impairments in Breast Cancer Survivors. PM&R.

[14] Stevens C.J., Hegel M.T., Bakitas M.A., Bruce M., Azuero A., Pisu M., Chamberlin M., Keene K., Rocque G., Ellis D. (2020). Study protocol for a multisite randomised controlled trial of a rehabilitation intervention to reduce participation restrictions among female breast cancer survivors. BMJ Open.

[15] Hahn E.A., Beaumont J.L., Pilkonis P.A., Garcia S.F., Magasi S., DeWalt D.A., Cella D. (2016). The PROMIS satisfaction with social participation measures demonstrated responsiveness in diverse clinical populations. J. Clin. Epidemiol..

[16] Hahn E.A., Devellis R.F., Bode R.K., Garcia S.F., Castel L.D., Eisen S.V., Bosworth H.B., Heinemann A.W., Rothrock N., Cella D. (2010). Measuring social health in the patient-reported outcomes measurement information system (PROMIS): Item bank development and testing. Qual. Life Res..

[17] Medidata COVID-19 and Clinical Trials: The Medidata Perspective. https://www.medidata.com/wp-content/uploads/2021/06/COVID19-Response4.0_Clinical-Trials_20200504_v3.2-1.pdf.

[18] Rentscher K.E., Zhou X., Small B.J., Cohen H.J., Dilawari A.A., Patel S.K., Bethea T.N., Van Dyk K.M., Nakamura Z.M., Ahn J. (2021). Loneliness and mental health during the COVID-19 pandemic in older breast cancer survivors and noncancer controls. Cancer.

[19] Tabaczynski A., Bastas D., Whitehorn A., Trinh L. (2023). Changes in physical activity and associations with quality of life among a global sample of cancer survivors during the COVID-19 pandemic. J. Cancer Surviv..

[20] Gurgel A.R.B., Mingroni-Netto P., Farah J.C., de Brito C.M.M., Levin A.S., Brum P.C. (2021). Determinants of Health and Physical Activity Levels Among Breast Cancer Survivors During the COVID-19 Pandemic: A Cross-Sectional Study. Front. Physiol..

[21] Skivington K., Matthews L., Simpson S.A., Craig P., Baird J., Blazeby J.M., Boyd K.A., Craig N., French D.P., McIntosh E. (2021). A new framework for developing and evaluating complex interventions: Update of Medical Research Council guidance. BMJ.

[22] Ejem D.B., Wechsler S., Gallups S., Khalidi S., Coffee-Dunning J., Montgomery A., Stevens C., Keene K., Rocque G., Chamberlin M. (2023). Enhancing Efficiency and Reach Using Facebook to Recruit Breast Cancer Survivors for a Telephone-based Supportive Care Randomized Trial During the COVID-19 Pandemic. J. Clin. Oncol. Oncol. Pract..

[23] Law M., Baptiste S., Carswell A., McColl M.A., Polatajko H., Pollock N. (2014). Canadian Occupational Performance Measure.

[24] Dimidjian S., Barrera M., Martell C., Munoz R.F., Lewinsohn P.M. (2011). The origins and current status of Behavioral Activation Treatments for depression. Annu. Rev. Clin. Psychol..

[25] Mundt J.C., Marks I.M., Shear M.K., Greist J.H. (2002). The Work and Social Adjustment Scale: A simple measure of impairment in functioning. Br. J. Psychiatry.

[26] Callahan C.M., Unverzagt F.W., Hui S.L., Perkins A.J., Hendrie H.C. (2002). Six-item screener to identify cognitive impairment among potential subjects for clinical research. Med. Care.

[27] American Occupational Therapy Association (2020). Occupational Therapy Practice Framework: Domain and Process.

[28] Lyons K.D., Svensborn I.A., Kornblith A.B., Hegel M.T. (2015). A content analysis of recovery strategies of breast cancer survivors enrolled in a goal-setting intervention. OTJR Occup. Particip. Health.

[29] Szanton S.L., Xue Q.-L., Leff B., Guralnik J., Wolff J.L., Tanner E.K., Boyd C., Thorpe R.J., Bishai D., Gitlin L.N. (2019). Effect of a Biobehavioral Environmental Approach on Disability Among Low-Income Older Adults: A Randomized Clinical Trial. JAMA Intern. Med..

[30] Gitlin L.N., Winter L., Dennis M.P., Hodgson N., Hauck W.W. (2010). A biobehavioral home-based intervention and the well-being of patients with dementia and their caregivers: The COPE randomized trial. Jama.

[31] Bachiochi P.D., Weiner S.P., Rogelberg S.G. (2004). Qualitative data collection and analysis. Handbook of Research Methods in Industrial and Organizational Psychology.

[32] Patton M.Q. (1999). Enhancing the quality and credibility of qualitative analysis. Health Serv. Res..

[33] Ernst M., Niederer D., Werner A.M., Czaja S.J., Mikton C., Ong A.D., Rosen T., Brähler E., Beutel M.E. (2022). Loneliness before and during the COVID-19 pandemic: A systematic review with meta-analysis. Am. Psychol..

[34] Lucchini L., Centellegher S., Pappalardo L., Gallotti R., Privitera F., Lepri B., De Nadai M. (2021). Living in a pandemic: Changes in mobility routines, social activity and adherence to COVID-19 protective measures. Sci. Rep..

[35] Khalifa S.A.M., Swilam M.M., El-Wahed A.A.A., Du M., El-Seedi H.H.R., Kai G., Masry S.H.D., Abdel-Daim M.M., Zou X., Halabi M.F. (2021). Beyond the Pandemic: COVID-19 Pandemic Changed the Face of Life. Int. J. Environ. Res. Public Health.

[36] Roben C.K.P., Kipp E., Schein S.S., Costello A.H., Dozier M. (2022). Transitioning to telehealth due to COVID-19: Maintaining model fidelity in a home visiting program for parents of vulnerable infants. Infant Ment. Health J..

[37] Naylor M., Hirschman K., Morgan B., McHugh M., Shaid E., McCauley K., Whitehouse C., Pauly M. (2021). Challenges and Strategies to Maintain Fidelity to the MIRROR-TCM Intervention During the COVID-19 Pandemic. Innov. Aging.

[38] Durosini I., Triberti S., Sebri V., Giudice A.V., Guiddi P., Pravettoni G. (2021). Psychological Benefits of a Sport-Based Program for Female Cancer Survivors: The Role of Social Connections. Front. Psychol..

[39] Robbins-Welty G.A., Mueser L., Mitchell C., Pope N., Arnold R., Park S., White D., Smith K.J., Reynolds C., Rosenzweig M. (2018). Interventionist training and intervention fidelity monitoring and maintenance for CONNECT, a nurse-led primary palliative care in oncology trial. Contemp. Clin. Trials Commun..

[40] Piamjariyakul U., Smothers A., Young S., Morrissey E., Petitte T., Wen S., Zulfikar R., Sangani R., Shafique S., Smith C.E. (2021). Verifying intervention fidelity procedures for a palliative home care intervention with pilot study results. Res. Nurs. Health.

[41] Lyons K.D., Erickson K.S., Hegel M.T. (2012). Problem-solving strategies of women undergoing chemotherapy for breast cancer. Can. J. Occup. Ther..

[42] Lyons K.D., Newman R., Adachi-Mejia A.M., Whipple J., Hegel M.T. (2018). Content Analysis of a Participant-Directed Intervention to Optimize Activity Engagement of Older Adult Cancer Survivors. OTJR Occup. Particip. Health.

[43] Patel A.V., Friedenreich C.M., Moore S.C., Hayes S.C., Silver J.K., Campbell K.L., Winters-Stone K., Gerber L.H., George S.M., Fulton J.E. (2019). American College of Sports Medicine Roundtable Report on Physical Activity, Sedentary Behavior, and Cancer Prevention and Control. Med. Sci. Sports Exerc..

[44] Cannioto R.A., Hutson A., Dighe S., McCann W., McCann S.E., Zirpoli G.R., Barlow W., Kelly K.M., DeNysschen C.A., Hershman D.L. (2021). Physical Activity Before, During, and After Chemotherapy for High-Risk Breast Cancer: Relationships With Survival. J. Natl. Cancer Inst..

[45] Littman A.J., Tang M.T., Rossing M.A. (2010). Longitudinal study of recreational physical activity in breast cancer survivors. J. Cancer Surviv..

[46] Knell G., Robertson M.C., Dooley E.E., Burford K., Mendez K.S. (2020). Health Behavior Changes During COVID-19 Pandemic and Subsequent “Stay-at-Home” Orders. Int. J. Environ. Res. Public Health.

[47] Acito M., Rondini T., Gargano G., Moretti M., Villarini M., Villarini A. (2023). How the COVID-19 pandemic has affected eating habits and physical activity in breast cancer survivors: The DianaWeb study. J. Cancer Surviv..

[48] O’Sullivan R., Burns A., Leavey G., Leroi I., Burholt V., Lubben J., Holt-Lunstad J., Victor C., Lawlor B., Vilar-Compte M. (2021). Impact of the COVID-19 Pandemic on Loneliness and Social Isolation: A Multi-Country Study. Int. J. Environ. Res. Public Health.

[49] Martell C.R., Dimidjian S., Herman-Dunn R. (2022). Behavioral Activation for Depression: A Clinician’s Guide.

